# Supplementary material for: Patterns of Intron Gain and Loss in Fungi
Source: PLoS Biol. 2004 Nov 30;2(12):e422. doi: 10.1371/journal.pbio.0020422 (PMC532390; doi:10.1371/journal.pbio.0020422)
Supplement: Table S1 — Also available at http://genes.mit.edu/NielsenEtAl/. (4.3 MB ZIP). [file pbio.0020422.st001.zip › NielsenEtAl/html/509.html]

AN4517.1.NCU00010.1.MG05190.1.FG05527.1


```
 CLUSTAL W (1.82) Multiple Sequence Alignments - Introns Inserted


Sequence 1: NCU00010.1	1053 aa
Sequence 2: MG05190.1	950 aa
Sequence 3: FG05527.1	1014 aa
Sequence 4: AN4517.1	920 aa
Alignment Length: 1107 aa
Number Identitical Residues: 340 aa
Alignment Score (without introns) 17297


MG05190.1 	--------MTEGSFGR-----ASRRTFRG----ATTLIVLSFAANAAN------------
NCU00010.1	--------MDALKSGRGFQPSLAEVVFRNSQLFYPVILLFAFIISAGIHSIVTSKTEEEV
FG05527.1 	MASADDIATTPLRGAARDDLPLADYILRKTQYLYALILLTAFISGAAWYSVANSKKQEDQ
AN4517.1  	--------MARHNVMRG-----SLEYLRIG---YPILVLVVFAAAFVANSIVTARTANQN
          	                      :   :*      . :::  *       :  .: . .. 

MG05190.1 	LAAQVMANADRLLPKT----------------------------------SYIAD-----
NCU00010.1	VAPTIKGPGGKPLPFTKRRREHHDIQAPF-VHNGDVAKTVFQYLSAGLVLSFVANGAAIA
FG05527.1 	VHSTIKGPGGKPLPITKTKSTLADGDRKIGPGFGRTAKNVFRYLAFVVFSTYVGNAGFMF
AN4517.1  	PSASQTGPGGRPLPKRSRSTVAVVKAN---QKFSQNAKLCFRLLSVVILVTLVAEAAVTV
          	  .   . ..: **  .                .  :.      :     : :.:..   

MG05190.1 	----------KPPQQ--WWCREESF0VYVVGTGSLYGYVLLTMFDWKDSPNFVHFLVWSV
NCU00010.1	LHAVSARGVKGEVGD--WWCDEQRI0VYVAGSAFFYVYVLITLFDWEHSPNIVHFITWVL
FG05527.1 	YHAFWFEDPYNWSQEGLPWAGEWSV0VHVTGALFFYLYILISLFDWRKGPNIVHFVVWIL
AN4517.1  	AHVMVAR-----SEQ--WWCGQAVV0IYVVGSFFDYAIILVSLLDTDPSPTFAQFVPWLV
          	              :   *. :  . ::*.*:   *  :*::::*   .*.:.:*: * :

MG05190.1 	SLVTDSVVLGALFARVSESYTVTS------NETLTGPAGNDIADLAIGAFRVANLIGVLA
NCU00010.1	GLGGEIVVLLSFALIVTGSHYSNFGAQDPIPKQTDGLDGWDLVDLSIGAVRILIVSIMVF
FG05527.1 	GFTGEVVIFSTTAVAAADCHFFRSVRNSDSGKDGNCVDYWTKVDLVFYFVRILHLLALIG
AN4517.1  	AAPLEMVILALSVSIYSRPHHEPAVGAPTGGKLRGGITLWEAAGVGCNSIRLLALLMLVM
          	.   : *::       :  :           :          ..:    .*:  :  :: 

MG05190.1 	LYLSLAGRRTFTVAEKREQEAETQPQSQSQVQNRRRSGSRRNRENGNDETTPLLGQRQ--
NCU00010.1	TYAALTTRKCIKEKQRYDEEASHS---------------------DSDESSPLLNG----
FG05527.1 	VFSLAWIRKARGLDDRKIEEAHPA------------------------ESTPLLHGHRRS
AN4517.1  	LYTCSSLRSPSVARKEARMNGDGP-----------------------NETTGLLRP----
          	 :     *      ..   :.                          .*:: **      

MG05190.1 	TSQNGYVAMNGNGRDRENRRSSQNGTANAAQVSVNGQSVG--PNREAEAAFYRPNKLPHK
NCU00010.1	SHPNYHTGSNRGNDRRLPRTDSGFGSENGAGEAGCQQ-------RDAEAAFYRPQKLPHK
FG05527.1 	YDTNGQLANGHTPNGREPNSRETNGRRGRGRTISNAHKTPSFPKKEEPTAFYRPEKLPHK
AN4517.1  	GTENGHAYG------------STNGAHG---------------DHKPADPWVRPTTQPST
          	   *                 .  *  .               .:.   .: ** . * .

MG05190.1 	TWWEYARGYSLFFPYLWPRDSRNLQLVVLLCFIILIFQRVVNVIVPVQTGRLVDRLQESL
NCU00010.1	TWFEYCRGYSVFFPYLWPANSLKLQGIILVCFVLVLLQRFVNIAVPAQMGRVTDALEPKD
FG05527.1 	TWWEYFKGYSLFFPYLWPKDSVKLQINVLICFILVMMQRLVNVAVPMAVKQVVNDLEEVI
AN4517.1  	SWWEYLSGYSLFFPYLWPSKSRRLQLVVLVCFLLIILQRVVNVLVPLQVGIITNKLSKQG
          	:*:**  ***:******* .* .**  :*:**:::::**.**: **     :.: *.   

MG05190.1 	DAVDGR------TMPWRDLALLIGTKILQGQSGLLGSIRAILWIPVSQYCYRALTSAAFE
NCU00010.1	GVFK---------MPWMQVGLLVLYKLLQGPSGLLGSLRSILWIPVSQHTYRALTTAAFE
FG05527.1 	KLVKDGERLSMDNFPLKDFMILGLLWILQGQSGLLGSLRSILWIPVSQYSYRGLTTAAFN
AN4517.1  	DEFR---------VPWFEICLYILFRWLQGNQGLIGSLRSTLWIPVSQYSYMELSTAAFE
          	  .          .*  :. :      *** .**:**:*: *******: *  *::***:

MG05190.1 	HVHSLSLDFHLSKRTGEVLSALNKGASINQFLEQVTFQVFPTLVDLFIAIGCFGWFFNSV
NCU00010.1	HVHSLSLDFHLGKRTGEVLSALNKGASINQFLEQTTFQVAPMLIDLFIAIIFFYVKFGPM
FG05527.1 	HVHSLSLDFHLSKRTGEVLSALNKGSSINQFLEQVTFQVFPMLFDLFLAISVFYYQYGPL
AN4517.1  	HVHGLSLDFHLGKKTGEVLSALSKGSSINTFLEQVTFQVVPMLVDLCVAIVYFLVALDAY
          	***.*******.*:********.**:*** ****.**** * *.** :**  *    .. 

MG05190.1 	YAVCVSVMTFAYLFLTVRMAATRADQRREMVNADREEEAVK~NDSIVSYETVKYFNAEDF
NCU00010.1	YALFVSVITFYYLFLTIRMAATRADQRRDMVNADREEEAVK~NDSITSYETVKYFNAEKY
FG05527.1 	YAEINLVDTCWYLYMTIKMASTRADQRREMTNADREEEAVK~NDSISSYETVKYFNAEEF
AN4517.1  	YALVVTIVTFCYLYVTIRMAQWRAEIRRQMVNSSRQEDAVK2NDSMVSYETVKYFNAEEY
          	**    : *  **::*::**  **: **:*.*:.*:*:*** ***: ***********.:

MG05190.1 	EFNRYRRAIRNYQSAEANVTWGINNMNICQNLVFMVGMLLLLIIGAYDVVIGARKIGDFA
NCU00010.1	EFARYRNAILNFQAAEAKVTFGMNHMNMCQSMVFMCGFLVIMMTCAYQVSQGTRTLGEFV
FG05527.1 	ESRRYQSKVAVFQGAEAKVQMGMMMMNICQTVVFNLGRIIAALVCGWQVAMGLRTTGDWF
AN4517.1  	EFGRYRGAVSDFQRAEYHVLFSLNLMNTSQNTIFMLGLLIACFIAAYQVSLGQRDVGQFV
          	*  **:  :  :* ** :*  .:  ** .*. :*  * ::  :  .::*  * *  *:: 

MG05190.1 	MLITYLGQLQGPLNFFGTFYRTVQQAMISGERLLELFKISPTVVDRPGVRTLPP-SVGHI
NCU00010.1	SLITYLGQLQGPLNFFGTFYRTIQQAMISGERLLELFKIQPSVIDGPDVQDLAS-CSGHL
FG05527.1 	MVVSYLTQLQGPLNFFGTFYRTVQQAMISGERLLELFKIQPTVVDTPHAVPLGKDFQGHV
AN4517.1  	SLLTYMAQLQGPLNFFGTFYRSIQSALINSERLLELFREQPTVVDRPNARPLAV-CKGDI
          	 :::*: **************::*.*:*..*******: .*:*:* * .  *     *.:

MG05190.1 	RWNNVKFWYDGKRPALKDISFECKPGTTTAFVGESGGGKSTIFRLMFRYYNCHEGSIEVD
NCU00010.1	KWNNVGFSYDKRRPALHDLSFECKPGTTTAFVGESGGGKSTVFRLMFRYYNCQEGSIQMD
FG05527.1 	RWNNVSFSYDRRKPALRNISFECQPGTTTAFVGESGGGKSTLFRHMFRYYDCDEGSIELD
AN4517.1  	KFDNVEFSYDARKPALNGLTFHCEPGTTTALVGESGGGKSTVFRLLFRFYNSERGRILVD
          	:::** * ** ::***..::*.*:******:**********:** :**:*:...* * :*

MG05190.1 	GNDVKDLTIDSVRRAIGVVPQDTILFNESIMYNLRYANQDATDEQVYEACKSASIHDRIM
NCU00010.1	GHDVKDLTIDSVRRFIGVVPQDTILFNETLMYNLKYANPNATEEDVYDACRAAAIHDRIL
FG05527.1 	GRNVKDLTINSVREAIGVVPQDTTLFNETLMYNLKYARPSATDEEVFAACRAASIHDRIL
AN4517.1  	GHDVESITIDSLRRHIGVVPQDTVLFNETLMYNLKYANPSATDEDVYEACRASSIHDKIM
          	*.:*:.:**:*:*. ******** ****::****:**. .**:*:*: **::::***:*:

MG05190.1 	SFPEGYNTKVGERGLRLSGGEKQRVAIARLILKQPRIIMLDEATSALDSETEQQIQSQLI
NCU00010.1	SFPDGYATKVGERGLRLSGGEKQRVAIARTILKNPQIIMLDEATSALDGETEQKIQSKLI
FG05527.1 	SFPDQYNTQVGERGLRLSGGEKQRVAIARTILKDPRIIMLDEATSALDSHTEQEIQDNVW
AN4517.1  	SFPDGYNTKVGERGLRLSGGEKQRVAIARTILKNPRIILLDEATAALDTETEEHIQGALS
          	***: * *:******************** ***:*:**:*****:*** .**:.**. : 

MG05190.1 	NGNHLGQDRTLLIIA2HRLSTITHADQIIVLRAGMIAEKGTHSELLKLNGKYASMWEKQS
NCU00010.1	SGN-FGEGRTLLIIA2HRLSTITHADQIIVLHAGTMVEKGTHQELLALGGRYASMWEKHC
FG05527.1 	S---IGQGRTLLIIA2HRLSTITHADQIVVLNNGTIVEKGTHEELLTAKGRYASMWEKQI
AN4517.1  	T---LSRGRTMLVIA~HRLSTITTADRILVLSEGKVTESGTHDQLLAMKGRYASMWRKQI
          	.   :...**:*:** ******* **:*:**  * :.*.***.:**   *:*****.*: 

MG05190.1 	KAERAALEARDAARRAEKLARQAKFAKASGKQQDHTDDSDGCYGN----TSAGSSTVLQA
NCU00010.1	RAERAAEHALRATLKAQKLLSRANLSRDDDHSDGDSMASSAILQTGLKSPTAGTSTPDNE
FG05527.1 	RAERALDVAREAQMKAAKAVRKAKMGIK--RNSDAAIDSYDGLGS----PGSLSGTGSQG
AN4517.1  	RAQKAAAEAQVLQDRAQRLR------------------------------SASTSAAAED
          	:*::*   *     :* :                                 : :.:  : 

MG05190.1 	TNALSED--SSTSDDDT-----------HKKPPNDNK---TTNPSPPPDL----------
NCU00010.1	SDTTSHRGTSSHSDDDSSASVSGSEGTLHDEASDDSHEEDNHNSAYDPRIAAIQPIAGRR
FG05527.1 	HGDDTHTSSSSSSDTESS----------HSEEHSSEH---KRK-----------------
AN4517.1  	S--------SSQSDEDRN----------TNRGPNANRQAQFHHP----------------
          	         ** ** : .           ..  . .:. .  :.                

MG05190.1 	------------------------------
NCU00010.1	DNGQPLSASYVSRSGLSSSCSLPRRVRAST
FG05527.1 	------------------------------
AN4517.1  	------------------------------
          	
```
